# Critical activities for successful implementation and adoption of AI in healthcare: towards a process framework for healthcare organizations

**DOI:** 10.3389/fdgth.2025.1550459

**Published:** 2025-05-16

**Authors:** Monika Nair, Jens Nygren, Per Nilsen, Fabio Gama, Margit Neher, Ingrid Larsson, Petra Svedberg

**Affiliations:** ^1^ School of Health and Welfare, Halmstad University, Halmstad, Sweden; ^2^ Department of Health, Medicine and Caring Sciences, Linköping University, Linköping, Sweden; ^3^ School of Business, Innovation and Sustainability, Halmstad University, Halmstad, Sweden

**Keywords:** artificial intelligence, implementation, adoption, deployment, process, framework, healthcare

## Abstract

**Introduction:**

Absence of structured guidelines to navigate the complexities of implementing AI-based applications in healthcare is recognized by clinicians, healthcare leaders, and policy makers. AI implementation presents challenges beyond the technology development which necessitates standardized approaches to implementation. This study aims to explore the activities typical to implementation of AI-based systems to develop an AI implementation process framework intended to guide healthcare professionals. The Quality Implementation Framework (QIF) was considered as an initial reference framework.

**Methods:**

This study employed a qualitative research design and included three components: (1) a review of 30 scientific articles describing differences empirical cases of real-world AI implementation in healthcare, (2) analysis of qualitative interviews with healthcare representatives possessing first-hand experience in planning, running, and sustaining AI implementation projects, (3) analysis of qualitative interviews with members of the research group´s network and purposively sampled for their AI literacy and academic, technical or managerial leadership roles.

**Results:**

The data were deductively mapped onto the steps of QIF using direct qualitative content analysis. All the phases and steps in QIF are relevant to AI implementation in healthcare, but there are specificities in the context of AI that require incorporation of additional activities and phases. To effectively support the AI implementations, the process frameworks should include a dedicated phase to implementation with specific activities that occur after planning, ensuring a smooth transition from AI's design to deployment, and a phase focused on governance and sustainability, aimed at maintaining the AI's long-term impact. The component of continuous engagement of diverse stakeholders should be incorporated throughout the lifecycle of the AI implementation.

**Conclusion:**

The value of this study is the identified processual phases and activities specific and typical to AI implementations to be carried out by an adopting healthcare organization when AI systems are deployed. The study advances previous research by outlining the types of necessary comprehensive assessments and legal preparations located in the implementation planning phase. It also extends prior understanding of what the staff's training should focus on throughout different phases of implementation. Finally, the overall processual, phased structure is discussed in order to incorporate activities that lead to a successful deployment of AI systems in healthcare.

## 1 Introduction

Artificial intelligence (AI) applications are increasingly being developed and tested in healthcare settings, enabled by enhanced analytical capabilities and advancements in computational technologies. These advancements allow for the processing of complex datasets and support from sophisticated AI-based applications for improvements in healthcare processes and health outcomes (1, 2).

However, previous research has highlighted the complexity of implementing AI in healthcare (3). The deployment of AI in healthcare is inherently more complex compared to sectors like manufacturing, retail, and finance, where standardized processes, lower regulatory constraints, and reduced risks to human life enable more straightforward implementation (4). Additionally, industries outside healthcare benefit from relatively easier access to large, high-quality datasets for training AI models, further facilitating adoption.

Healthcare, on the other hand, involves intricate interactions between human providers, patients, and technology, where the risks are exceptionally high, and any compromise in patient safety or quality of care must be rigorously avoided. The integration of AI introduces additional challenges and uncertainties related to clinicians' liability in using the AI model outcomes in decision-making or neglecting them, autonomy of the patient and clinician, explainability, ethics, and the need for oversight and quality control (5–9). Compared to other sectors, healthcare faces structural barriers to technological adoption, encompassing technological, individual, social, and organizational domains (6, 10–12). For AI specifically, these barriers encompass challenges related to data, methodologies, technology, regulations and policies, human factors, environmental conditions, and organizational structures (6–9). For example, data quality is often compromised for the sake of faster workflows creating challenges in obtaining high-quality data for AI model training. Consequently, clinicians and patients may lack trust in the AI models for decision-making, a situation further burdened by unclear boundaries of liability. Also, organizational structures and different data storage practices lead to difficulties in combining the data.

Clinicians have recognized a lack of structured guidelines to navigate these complexities, ensuring that AI-based applications are implemented in a more predictable manner that enhances, rather than hinders, clinical workflows and patient outcomes (13, 14). Healthcare leaders emphasize the importance of considering implementation early in the development process, moving beyond the technology development and recognizing the significant challenges associated with practical implementation (8). Policymakers are also understanding the need for comprehensive guidelines to ensure that AI implementations comply with stringent regulatory (15) and ethical standards (16).

However, research indicates that the implementation of AI-based applications in healthcare has often lacked standardized and structured methodologies (2, 17). Despite growing attention to the challenges associated with implementation, there remains a significant need for empirical, experience-based research to identify strategies, processes, and methods that can effectively address these barriers (18, 19). Such research has the potential to inform the development of guidelines to support the integration of AI in healthcare.

Several frameworks have emerged for research and development purposes specifically addressing AI, such as the research-based evaluation framework for AI-based decision-support systems (20), the reporting guidelines for clinical trial reports for interventions involving AI (21), the provisional AI implementation framework (19), International consensus guideline for trustworthy and deployable AI in healthcare (22), the adoption of AI in the Healthcare Industry Model (23), and the organizational governance framework for AI adoption (24). Several studies have addressed elements crucial to implementation such as managerial, cultural, individual, and technological challenges, with some insights into the implementation process (2, 9, 17). However, studies have not been sufficiently detailed to provide sufficient processual support that could guide AI implementations to facilitate their integration and use in practice.

For a more standardized approach to AI implementation, implementation science emphasizes the importance of comprehensive planning from the outset (25). Such planning involves creating conditions that are favorable for implementation and for carrying out the process in a structured, well-planned, and orderly manner. Process frameworks are used in implementation science to provide guidance for implementation by outlining key considerations and activities that need to be undertaken before, during and after the implementation process (26).

The Quality Implementation Framework (QIF) (27) is a widely recognized process framework within implementation science, known for its adaptability and applicability across diverse contexts, including healthcare (28–31). Aiming at increasing quality of implementations, QIF's creators Meyers et al. Have built the QIF on 25 theories, models, and frameworks across multiple research and practice areas. The resulting QIF framework contained 14 activities clustered into a 4-phase temporal sequence (27). Its structured and comprehensive approach offers detailed guidance on key activities and considerations necessary for successful implementation, making it a promising candidate for structuring the implementation of AI-based applications in healthcare. Unlike many other implementation process frameworks, which often provide more generalized guidance on translating research into action, the QIF stands out for its practical specificity and focus on actionable steps (28). The structure of the QIF includes four phases with detailed activities in each. The first phase recommends considering the host setting and examining how the innovation and the context fit each other. The second phase is focused on structuring the implementation process and assigning personnel. The third phase guides towards a necessity of support and feedback mechanisms for the implementation process. The final fourth phase is dedicated to learning and reflection to create capabilities leading to improved future implementations in the organization.

This study constitutes an integral part of a broader project (18) aimed to develop, test and evaluate a framework to guide the implementation of AI-based applications in healthcare. This paper focuses on the framework development part (32), with the specific aim to explore the activities typical to implementation of AI-based systems for developing an AI implementation process framework intended to guide healthcare professionals, leaders, and decision makers who plan, lead, or are involved in the implementation processes. To achieve this aim, the QIF was considered as an initial reference framework.

## 2 Materials and method

### 2.1 Design

**Figure 1:**
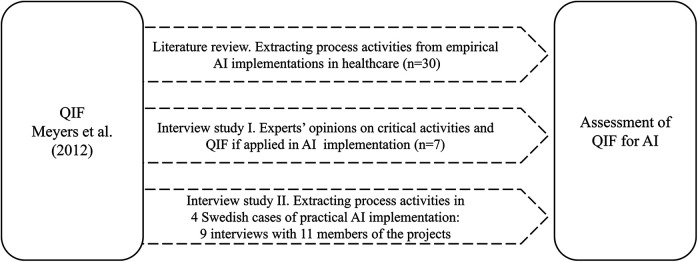
Study design.

This study employed a qualitative research design (33), utilizing two complementary methods for data collection and analysis: a review of scientific articles and analysis of qualitative interviews (Figure 1). The literature review on multiple examples of real-world AI implementations in healthcare practice provided a broad overview of process steps and lessons that comprised AI implementation within different types of AI systems, clinical areas and geographies as well as the lifecycle of AI systems—from development to deployment and adoption. The qualitative interviews had a two-fold goal: first, to collect direct individual reflections on experiences in the AI implementation process, and second, to reflect on what structure and content of the QIF may contribute to inform a future process framework specifically adapted for the implementation of AI in healthcare. The integration of these methods and diverse data sources enabled data triangulation, which increased the quality and credibility of research findings (34) and provided a more rounded understanding of how AI implementations are and should be conducted in practice. The knowledge generated from this study will inform future methodological developments to support AI implementations. The interviews performed in this study did not include sensitive personal information of the participants, and therefore, according to the Swedish legislation on research ethics, an ethical approval was not required.

### 2.2 Sample and data collection

#### 2.2.1 Literature review

The literature review started with identifying the scoping reviews and systematic literature reviews (in PubMed) on empirical studies of AI implementation in healthcare. A set of references to the empirical studies was extracted from these reviews resulting in 38 articles published between 2011 and 2023, covering various countries of origin [the articles' search strategy is described in (9)]. Earlier articles were excluded from the scope of the present study due to the rapid pace of technological advancement and the aim to focus on contemporary AI solutions. The selected articles were reviewed by title, abstract, and full text when needed to identify ones that contained implementation process data and resulted in 30 articles included. The remaining 8 articles had no focus on the implementation process and were excluded from this review.

Most of the articles included represented AI systems developed, tested, and gone through real-world implementations within healthcare organizations discussing process steps and challenges during the different stages of AI system's lifecycle, from development to adoption. Of the total of 30 articles, 14 articles (47%) examined AI applications in treatment, 13 articles (43%) explored AI applications in diagnosis, and 3 articles (10%) investigated AI applications in prevention (Supplementary Material S1). For simplicity, the categorization of the articles was made mutually exclusive, with each article classified under the most predominant aspect as determined by the author's understanding. However, some articles may overlap and fit into multiple categories. The data extracted from these included articles were process steps, activities, involved stakeholders, and lessons learned.

#### 2.2.2 Interview study I

Seven interviews were conducted between September–November 2023 with experts with the aim of collecting their perceptions of the critical activities in the AI implementation process and appropriateness of QIF for use in real world practice during AI implementations in healthcare (Table 1). The participants were explained the study purpose and the procedure, and that the project does not handle any sensitive personal data. They were also informed about the possibility to access their anonymized data and provide corrections, if needed. Then, the participants provided verbal consent to participate and to express personal experiences and perceptions. The interview questions related to how can a detailed guide support real-world implementations of AI in healthcare, and their impressions of the QIF in connection with the implementation of AI, its potential benefits and disadvantages, and the level of detail (the interview guide is provided in Supplementary Appendix S2). Participants were members of the research group´s network and purposively sampled for their AI literacy and academic, technical or managerial leadership roles. The data was collected via video communications (averaging 30 min, with a range of 10–50 min) and e-mail conversations, that were sometimes repeated to ask for more detail or to clarify written feedback.

**Table 2:** Cases and participants in the interview study II.

Case	AI system	Responsibility in AI implementation project
Case 1	AI-based decision support system 1	Clinical champion—implementation lead
Case 2	AI-based decision support system 2	Clinical champion
		Implementation project lead
		IT architect
Case 3	AI-based patient monitoring system	Group interview: Project owner, digitalization lead, strategist
Case 4	AI-based system facilitating clinical administration	Implementation project lead
		Regional implementation lead
		IT architect
		Communications manager

#### 2.2.3 Interview study II

Given the focus on understanding the process of real-world AI implementation in practice, and aiming to deep-dive into how the sustainable change of practice was achieved, persons possessing first-hand experience in planning, running, and sustaining such projects were purposively recruited (35) from four cases of AI implementation that had AI systems procured and in use in practice. To achieve a rounded view of the process and variability between clinical areas and the types of AI, these participants represented positions within different cases carried out in large healthcare organizations from different geographical regions in Sweden (Table 2). Two of the projects were AI-based decision-support systems in different clinical areas, one project was an AI-based patient monitoring system, and one project was an AI-based system facilitating clinical administration. These cases were selected using convenience sampling (36) due to a scarcity of available cases where AI systems have been integrated into routine practice in healthcare. The interviews were conducted in May–September 2024 with 11 professionals in 9 interviews (Table 2). The participants were explained the study purpose and the procedure, and that the project does not handle any sensitive personal data. They were also informed about the possibility to access their anonymized data and provide corrections, if needed. Then, the participants provided verbal consent to participate and to express personal experiences and perceptions. The interview used open-ended questions with a focus to discern implementation process activities, their sequence, involved stakeholders, and lessons learned. The interviewees were asked questions like “Why and how did the implementation process start, who initiated the project, what was the trigger?”, followed by “How was this activity performed, who were involved?”, then directing the conversation to “What activities happened next?” until the sequence of activities was identified. Each interview lasted between 1.5 and 3 h, totaling 15 h.

**Table 1:** Participants in the interview study I.

Expert	Role	Interview date
Expert 1	Professor, AI researcher, University in Sweden	October 2023
Expert 2	Academic coordinator of AI research program, University in Sweden	October 2023
Expert 3	Primary health care specialist and academic partner at University in Sweden	September 2023
Expert 4	Professor, Senior physician, Sweden	October 2023
Expert 5	Senior executive, Sweden	November 2023
Expert 6	Senior Data Scientist, AI Competence Center, Sweden	October 2023
Expert 7	Innovation project managerSweden	October 2023

### 2.3 Data analysis

The data on processual activities, decisions, involved stakeholders, challenges and lessons learned from every empirical study selected during the literature review were listed for mapping an individual process of every case. The studies provided varying level of detail, often with an extensive focus on a few activities depending on the purpose of the study or on the issues the authors had aimed to highlight. Then the data were deductively mapped onto the steps of QIF using direct qualitative content analysis (31) aiming to compare the processes described in the studies and the QIF. Data not fitting onto the QIF but reflecting realities of the AI implementation process were registered separately.

The interview transcripts from the interview study I detailing the perceptions of experts about QIF were reviewed using direct qualitative content analysis (33). Insights were labelled using the numbering of the QIF steps. The insights not fitting onto the QIF but reflecting realities of the AI implementation process were registered separately. This allowed to determine areas of alignment and gaps between QIF and AI implementation practice and to identify potential modifications or extensions of the QIF.

To extract the AI implementation process from the case interviews (interview study II), the interview transcripts were reviewed aiming to familiarize with the context, to identify start and end points, key actions, decisions, and involved parties and their responsibilities. Then the data were listed for mapping an individual process of every case. Then the data were deductively mapped onto the steps of QIF using direct qualitative content analysis (33). This allowed for comparisons between the processes of the case and the QIF. Data not fitting onto the QIF but reflecting realities of the AI implementation process were registered separately.

Three pairs of co-authors evaluated the data independently, discussed and reached a consensus through a number of iterations (37). Data not fitting onto the QIF were included in two different ways and later processed during the data analysis. First, data (condensed quotes) within the same area as respective QIF steps were documented in a separate column next to each step of QIF (Supplementary Material S3). Second, the data on activities of the practical AI implementation process that QIF does not cover were documented, analysed and summarized separately in the paragraph “Limitations of QIF for AI implementation” below.

## 3 Results

The data analysis showed that all the phases and steps in QIF are relevant in the context of AI implementation in healthcare practice. The processual activities extracted from the literature studies and the interviews representing the AI implementation cases have reflected the QIF process while providing additional details that should be further incorporated to enhance AI specifics. The expert interviews have also validated that QIF can provide a sound conceptual basis for describing and guiding the AI implementation process, although the specificities of the AI context would require some refinement in phases and steps and adding more details to effectively support it. Further, every step of QIF is discussed in the context of AI based on the data outlined in Supplementary Material S3, ending the section with the limitations of QIF for AI implementation. Definitions of the Cases 1–4 as well as Experts 1–7 referred to below can be found in Tables 1, 2.

### 3.1 QIF in the context of AI implementation in healthcare

#### 3.1.1 QIF step 1—conducting a needs and resource assessment

According to QIF, this step recommends a thorough understanding of the problem, its root causes, and the intended beneficiaries of the innovation. The data underscores the relevance of step 1 in AI implementation, where practitioners utilize data analysis to assess the magnitude of the problem and systematically review published research for evidence supporting AI's applicability to the problem (13, 38–42) Furthermore, the data analysis identified additional activities related to the needs assessment process relevant to AI implementation:

• Preparing a case demonstrating a clear need: what should be solved and why (Case 2). Such information is useful for managerial approvals, in communication activities and in change management.

#### 3.1.2 QIF step 2—conducting a fit assessment

According to QIF, this step recommends assessing how the innovation fits with the setting by considering identified needs, the organization's mission, values, strategy and priorities, as well as cultural preferences. The data confirms the necessity of step 2 for AI implementation, where practitioners investigate whether an internal development or purchasing of the AI system is aligned with the identified needs, the innovation strategy, institutional priorities, and identified needs as well as identifying what the added value would be (39, 40, 43–45). The data analysis identified additional activities related to the fit assessment process specific to AI implementation:

• Identifying if the AI system has a suitable regulatory certification (Expert 3, Case 3)
• Investigating relevance of the data that the commercial AI model was built on, possibly testing performance on own data (Expert 3, Expert 6)
• Investigating what are the conditions for retraining the model and for monitoring model's performance (Expert 6)
• Investigating ethical aspects of the AI model: bias, participation, integrity, demographics, etc. (Expert 3)
• Investigating impact on data security (Case 3)
• Analyzing potential risks for the users and the patients (Expert 2, Case 4)
• Assessing legal aspects of the solution and the collaboration with the vendors (Case 3, Expert 4).
• Assessing compatibility of the AI system with organization's processes (46).

#### 3.1.3 QIF step 3—conducting a capacity readiness assessment

According to QIF, this step recommends assessing if the organization has adequate resources, skills, and motivation to implement the innovation. The data indicates that step 3 is also relevant to AI implementation, where the practitioners assess the required resources and consider potential changes in staff's employment and roles (13, 39, 45), (Case 2, Case 4). The data analysis identified additional activities related to the capacity and readiness assessment relevant to AI implementation:

• Analyzing benefits vs. costs (43)
• Investigating the opportunity cost: what might be lost due to the introduction of AI, e.g., deskilling staff. (Expert 3)
• Analyzing possibilities for relocation or requalification of staff (Case 4)
• Investigating sufficiency of the technical environment in an organization (e.g., computing power) (Expert 6)

#### 3.1.4 QIF step 4—possibility for adaptation

According to QIF, this step recommends assessing whether the innovation should be modified to fit the setting and the target group, and how the changes would be documented and monitored during the implementation. The data indicates that step 4 is relevant to AI implementation, with practitioners focusing on identifying organizational constraints and determining if any changes or additional developments of the AI system, IT infrastructure, or workflows are necessary (13, 39, 43–49). The data analysis identified that conducting a local pilot is an important activity dedicated to understanding the local relevance of the AI system and necessary adaptations. The pilot should be set up considering the technical infrastructure, legal side, contracts, and workflows. Before the pilot, training should take place, and evaluation should be planned, including the collection of user feedback based on the pilot (Case 1, Case 3, Case 4).

#### 3.1.5 QIF step 5—obtaining explicit and implicit buy-in and approvals/permissions

According to QIF, this step includes achieving the buy-in by leadership, staff, communities, addressing organizational resistance, and recruiting innovation champions. The data confirms that step 5 is relevant to AI implementation, highlighting practitioners' efforts to demonstrate the added value of the system, to organize support from staff and leadership, and appoint champions (13, 39–41, 44–45, 48–55). The data analysis identified additional activities related to obtaining the buy-in specific to the AI implementation process:

• Involving clinicians in designing a user-friendly AI system and its user interface, in system's training and in contextualizing data representation (41)
• Addressing clinicians' legal liability questions and preparing the documents detailing clinicians' responsibilities in the context of the AI system (40, 44), (Expert 3), (Case 1, Case 2)
• Addressing algorithm's explainability, availability, quality and safety (45, 48)
• Using a thoughtful framing about AI in communication with the stakeholders (13)

#### 3.1.6 QIF step 6—building organizational capacity

According to QIF, this step includes investigating which aspects of the organization's infrastructure, skills, and motivation require enhancement to accommodate the innovation. Also, QIF specifically points out that these enhancements do not directly assist with the implementation but instead create a supportive environment for success. The data analysis underscores the relevance of step 6 to AI implementation, particularly highlighting practitioners' efforts to build relationships within the organization that enable better workflows related to the AI system (42). Although this step recommends investigating the capacity of the infrastructure, the analysis indicates that this activity should be planned earlier, for example, within the fit assessment in Step 2 in QIF.

#### 3.1.7 QIF step 7—staff recruitment/maintenance

According to QIF, this step involves recruiting staff responsible for the implementation process and for supporting the frontline staff. It also includes an assessment of whether the roles of staff might change. The data indicates that step 7 is relevant to AI implementation, with project leaders and teams being formed or newly recruited to support the practitioners in this process (13, 56). The data analysis identified additional aspects specific to AI implementation:

• Managing AI implementation might require new recruitment not only for the project's execution but also for later managing routine tasks involving the AI system (40, 42).
• Assessing potential changes in roles recommended in this step of QIF would be conducted earlier in the implementation process of AI, possibly during the organization's capacity assessment (Step 3 in QIF). Without such understanding, it is hard to determine whether and how the introduction of the AI system would add value, and it complicates striving for the buy-in defined in earlier steps (Step 5) and the ethical handling of staff.

#### 3.1.8 QIF step 8—effective pre-innovation staff training

According to QIF, this step includes teaching staff about the innovation and its values to enable them to apply the innovation. The data highlights the importance of step 8 to AI implementation, revealing that training is not limited only to the pre-innovation phase, but continuous throughout the next phases (13, 39, 42, 48, 49, 56–58), with each phase focusing on the following specific objectives:

• Pre-innovation training should focus on skills and competence building in the areas that enable and support the use of AI (50). Depending on the context, it could include, for example, cross-unit collaboration and communication, skills of training other people, empathy, attentive listening, and similar interpersonal competencies (50). In the pre-innovation phase, general technical AI competences should be developed, equipping the staff with conceptual, ethical, and legal knowledge about AI.
• During the implementation, training should concern the vision for change and the urgency for improvement, and the scientific and clinical basis for the specific AI system (42, 48, 49). Next, it is crucial to develop skills in the practical use of the specific AI system and the new workflows, highlighting the strengths and weaknesses of the AI model for trust enhancement (13).
• Post-implementation training intends to ensure sustainability of the new way of working and the AI system's use (39). Apart from on-boarding of the new staff that joined after the implementation, the post-implementation training could include case-based reviews for training purposes (49).
• Preparing quality training materials and formalized flowcharts for the users to follow as well as conducting the training through different phases, can be costly and this cost category should be considered before the start of the implementation (13), (Case 1, Case 3, Case 4).

#### 3.1.9 QIF step 9—creating implementation teams

In QIF, this step includes determining who in the organization would carry the responsibility for the implementation process and outcomes as well as who would be the supporting team. The data indicates that step 9 is relevant to AI implementation, with implementation project leaders and teams being formed or newly recruited (13, 40, 56), (Expert 7, Case 1, Case 2, Case 4).

#### 3.1.10 QIF step 10—developing an implementation plan

According to QIF, this step includes setting up tasks and timelines and foreseeing challenges that can hinder the implementation. The data confirms the necessity of step 10 for AI implementation, emphasizing that practitioners worked on setting clear goals, planning test phases and establishing a structured meeting schedule (13, 44). Additional actions specific to developing a plan for AI implementation were:

• Assigning an organizational owner of the AI system which would be responsible for the system's rollout, maintenance and changes in the future, as well as the related budget (Case 2)
• Creating a communication plan (Case 4)

#### 3.1.11 QIF step 11—technical assistance/coaching/supervision governance

This step in QIF is meant to address the practical problems arising during the implementation. The data indicates that step 11 is relevant to AI implementation, emphasizing the importance of creating robust support mechanisms. Practitioners concentrated on ensuring the availability of specialists, establishing IT support and chat systems and equipping staff with essential resources, including appropriate equipment and training materials (13, 52), (Case 1, Case 4). The data analysis did not identify any further activities specific to AI implementation.

#### 3.1.12 QIF step 12—process evaluation

This step in QIF concerns the evaluation of the implementation process; how it unfolds over time in light of the innovation being implemented, and how different individuals performed. The data has shown that step 12 is relevant to AI implementation, and underscores its relevance for practitioners to evaluate not only the AI implementation itself but also key factors such as barriers and facilitators to adoption, unintended social consequences, impact on clinical roles, responsibilities, and trust, and perceptions of evidence (13, 48). The data analysis identified additional evaluation aspects relevant to AI implementation:

• Assessing clinical and operational impact to demonstrate safety, efficacy, and added value (13, 39, 44, 48, 60), (Case 1)
• Assessing and monitoring users' engagement and AI system's usage (59), (Case 4)
• Evaluating effects on processes and staff caused through the new practice when using AI system (39, 60)
• Gathering and analyzing user feedback (13, 61)

#### 3.1.13 QIF step 13—supportive feedback mechanism

According to QIF, this step recommends creating a process and channels through which feedback on the implementation process could be communicated with those involved in the innovation. The data has shown that step 13 is relevant to AI implementation, focusing on gathering feedback and further requests for adaptations, identifying shortcomings or risks, and addressing the need for additional training. This feedback and insights were collected post-deployment of AI through governance activities through analyzing data and using different channels such as e-mail, web-based survey, or meetings with staff (13, 41, 45, 50, 57, 58, 62, 63), (Case 1). The data analysis did not identify any further activities specific to AI implementation.

#### 3.1.14 QIF step 14—learning from experience

According to QIF, this step recommends analyzing and reflecting upon lessons learned from the implementation process and sharing them with other interested parties. The data analysis underscores the relevance of step 14 to AI implementation, building on the premise that AI system's implementation is never finished and needs continuous monitoring, maintenance, and development to fit the business, particularly highlighting importance of such reflection and knowledge transfer in the situations where consultants were recruited for assisting in the implementation (Expert 1).

### 3.2 Limitations of QIF for AI implementation

The analysis revealed critical limitations in QIF when applied to AI implementation in healthcare, necessitating emphasis on two additional phases and important activities. These include a dedicated phase for the practical implementation activities that occur after planning, ensuring a smooth transition from design to deployment, and a phase focused on governance and sustainability, aimed at maintaining the AI's long-term impact. Additionally, the activity of continuous engagement of diverse stakeholders throughout the lifecycle of the AI implementation project is essential for its success. Incorporating these components into a dedicated framework is critical to support effective and sustainable deployment of AI in healthcare.

#### 3.2.1 Practical implementation activities after planning

The data underscores the importance of incorporating a phase specifically dedicated to the actual implementation activities that occur after planning. The data analysis revealed that during the AI-specific implementation phase, the actual changes in the local process workflows and protocols should be established. Their descriptions and all the related documentation should be updated by incorporating AI details, and those changes should be incorporated into the training materials (44, 46, 49–51), (Case 2, Case 4). Accordingly, existing roles and competences might require adaptations in the job descriptions which should also be necessary to include in the training (39, 41, 42). On the technological side, actual changes in the IT infrastructure of the organization should be implemented establishing the secure data management, building test and production environments (46, 51), (Case 2). The data analysis also underscores the importance of change management activities, such as continuous communication with staff, AI vendors and developers and conducting training of AI system's users addressing the system's technical specifics, the new workflows and roles (Case 2, Case 4). Finally, AI system's usage monitoring and compliance procedures should be established to follow up on the actual utilization of the system (13, 41, 45).

#### 3.2.2 Governance activities for ensuring sustainability of Ai system

The data highlights the need to incorporate a dedicated phase focused on governance and sustainability to ensure the ongoing maintenance and success of AI innovations. The data analysis revealed that the effective means for promoting sustainability of the AI system implemented is through creating a governance body or committee (13, 41, 45) that could include representatives from leadership, frontline staff, information technology and innovation specialists. Activities of the governance committee include pilot study, reorganizing the workflows, monitoring AI system's performance and effectiveness and a possible “model drift”, addressing concerns of staff collected through the feedback mechanisms, promoting and tracking usage of the system, developing reporting, monitoring for a proper insertion of patient records, providing post-implementation training, prioritizing and approving changes, and planning for further financing (13, 41, 45, 50), (Expert 6).

#### 3.2.3 Continuous engagement of diverse stakeholders

The data analysis revealed that engagement of different stakeholders spans throughout the lifecycle of the AI implementation project. The engagement should start at the ideation and problem formulation phase (46) and should be sustained throughout the implementation cycle, which is crucial for managing change (13, 40, 45, 46, 50, 55, 56, 62). Obtaining stakeholders' confirmation that the problem is relevant allows to consider a global view and to formulate clear use case at the onset of the implementation since different stakeholders might need different output from AI or have different interpretations; it also promotes their readiness for change (40, 45, 46). Throughout the implementation process, engaging the stakeholders can help in several ways. It includes obtaining scientific, theoretical and practical knowledge of the health area which is useful for technology professionals (50). Next, the stakeholders can help in defining the parameters of the targeted population, data features to train the model on, and to think through how the model could be deployed (41, 46). Later, they can report on the user experience and whether the AI model aligns with their needs (50). Moreover, the stakeholders can play a role in designing the evaluation of the AI model (41) and can participate in creating the training materials (13). Finally, early engagement assists in change management and training since clinicians develop intuition and gain experience that advances their ability to train others (13).

## 4 Discussion

This study aimed to explore the activities typical to implementation of AI-based systems for developing an AI implementation process framework intended to guide healthcare professionals. To achieve this aim, QIF was considered as an initial reference framework (26).

Previous research has indicated that a lack of stepped guidance and AI specificity in the presently available implementation frameworks can lead to patient safety risks, dissatisfaction of clinical staff, extra costs and delays in the AI system's deployment as demonstrated by previous research (13, 17, 39, 40). The key findings of this study are the identified processual phases and activities specific and typical to AI implementation projects to be carried out by an adopting healthcare organization when AI systems are deployed in routine practice. By reflecting upon the QIF, additional phases of implementation and governance were discerned, and different AI-specific activities were detected in connection to the QIF steps.

A significant finding of the study is that successful AI implementation necessitates comprehensive pre-implementation assessments. Some types of identified assessments align with the ones suggested by QIF, such as the in-depth analysis of the problem and stakeholder needs, assessing compatibility of the AI system with an organization's strategy, processes, IT infrastructure, and potential impact on patients, clinical roles, and the organizational culture. These aspects in need of assessment were also recommended by previous research discussing the innovation process or determinants in the context of digital health and health innovation (64–66). The present study identified additional types of recommended assessments in the context of AI and located them as necessary activities in the planning phase: investigating explainability of AI algorithm (45, 48), relevance of an AI model to the local data (Expert 3) and conditions for re-training the model (Expert 6), level of data security and protection (Case 3), risk-consequence analysis (Expert 2, Expert 3, Case 4), cost-benefit analysis (43), considering the opportunity cost (Expert 3), ethics and patient safety considerations (45, 48), (Expert 3). Furthermore, the present study identified several preparatory actions in the legal area not described by the previous research. In addition to the traditional activities like negotiating different contracts and agreements between the organization and the vendor and verifying whether the AI system is suitably certified (59), this study added activities like setting up agreements regarding the data flow and protection and defining the policy explaining clinicians' liability (40, 43, 44), (Expert 3).

The present study identified a number of important activities that could be added to the actual implementation phase. These implementation activities include creating new or updating and standardizing local procedures and clinical protocols integrating the AI system, changing or creating new job descriptions, adapting IT architecture and setting up secure data management, conducting training and continuously communicating with users (44, 46, 49–51, 57), (Case 2, Case 4). Previous research either did not emphasize them entirely (65) or has reflected upon elements of it through different determinants, barriers and facilitators (64). This study was not able to place these activities within the process view of QIF which indicates that a specialized framework for AI implementation process should integrate them in the future.

This study underscores the importance of incorporating creation of the organizational structures and processes for the post-implementation governance period (13, 41, 45) as an integral part of the AI implementation framework. In the “follow-up” stages of AI-implementation, these processes are even more important, because not only the continued human adherence guarantees the sustained use, but also the monitoring of the model's performance and patient safety. Notably, this crucial phase dedicated to governance of maintenance and sustainability of the AI systems is absent in existing implementation frameworks such as (26) and (64). While specialized frameworks and different national guidelines focusing on governance of AI are beginning to emerge (67), a process-oriented perspective remains lacking. This perspective should address several key questions: When and how should governance work commence? What practical organizational structures and processes can facilitate effective governance? What is the role of leadership in this context? How can various stakeholders (e.g., healthcare organizations and vendors) collaborate to ensure the sustained use of AI systems? Ultimately, how can we guarantee the enduring effectiveness and sustainability of AI governance? Addressing these critical questions is paramount to ensuring that governance, maintenance, and sustainability of AI systems remain a top priority for healthcare practitioners during the implementation process.

Further, other significant finding is the importance of prioritizing staff training throughout all phases of AI implementation, pre-, during, and post-implementation, to ensure success. These findings extend previous research that was limited to the overall content of the training for AI implementation (22, 67). This study emphasizes that pre-innovation training might be insufficient in the context of AI. It requires a continuous, iterative approach starting pre- and throughout the lifecycle of the AI system and should have a focus on the related clinical context and processes surrounding the AI system. In addition, specific competences and skills of staff should be developed prior to engaging in AI implementation (50). The study also revealed insights into the intensity of resources required for conducting effective training that enables staff to engage in using the AI system in their practice sustainably. The cost category related to continuous training and preparation of the training materials should be considered when evaluating the organization's capacity for conducting the AI implementation and in planning the costs (41), (Case 2).

Lastly, another crucial finding is that engagement of the stakeholders should span throughout all the phases of the AI implementation (13, 40, 45, 46, 50, 55, 56, 62) which corresponds to previous research highlighting the importance of stakeholder input and involvement (22, 64, 65). However, earlier implementation frameworks have not discerned the practical activities of stakeholder involvement through the processual perspective of different phases of implementation. During the preparatory phase, the stakeholders' role is to provide the subject knowledge, to confirm the relevance of the problem and the use case, to help define the target population and the parameters of the training data, and to envision the new workflows integrating the AI system (40, 41, 45, 46). The stakeholders should also be part of the AI system's piloting and evaluation activities and provide feedback (41). During the implementation phase, the stakeholders are involved in training as trainers or trainees and assist in communication and dissemination activities (13). During the governance and maintenance phase, the stakeholders can have roles in the AI system's governance committee, provide continuous feedback and ideas for improvement (13, 41, 45).

While this study offers valuable insights into the process of AI systems' implementation, several limitations exist. First, the dataset used for analysis does not include scientific publications published since 2024 or cases representing generative AI applications. While the present study provides a solid foundation for understanding the implementation process for AI systems, newer types of AI models might present additional important implementation practices not covered in this article. Further, the data collected through the interview studies with the representatives from AI implementation cases and the experts represent the Swedish context and might not depict some practices manifesting in other healthcare contexts. Moreover, the cases were selected using convenience sampling which might provide limited generalizability of the results and overrepresentation of certain types of AI systems. Also, access to interviewees in Case 1 was limited to the implementation leader. This limitation was partially addressed by conducting two interviews with the same person to obtain more details on the implementation process.

Future research should discern processual differences based on the typology of AI (for example, AI systems dedicated to improving administrative processes of healthcare organizations compared to diagnostic AI systems). Another potential research avenue could relate to exploring potential differences in implementation when AI models are developed internally at healthcare organizations and procured from external vendors. How can stakeholders be educated to ensure their proper engagement throughout all phases. The future research could also address practicalities behind different activities in the implementation process and how they influence the success of the implementation and its sustainability. Systematizing such knowledge would create a better understanding of the interconnections between the activities and would help understand their relative weight for success of the implementation.

## 5 Conclusion

The study aimed to explore the activities typical to implementation of AI-based systems for developing an AI implementation process framework intended to guide healthcare professionals. To achieve this aim, the Quality Implementation Framework was considered as an initial reference framework. The analysis revealed its gaps when applied to AI implementation in healthcare, necessitating the inclusion of additional phases and important components. These components include a dedicated phase for the practical implementation activities that occur after planning, ensuring a smooth transition from design to deployment, and a phase focused on governance and sustainability, aimed at maintaining the AI's long-term impact. Additionally, the component of continuous engagement of diverse stakeholders throughout the lifecycle of the AI implementation project is essential for its success. The identified processual peculiarities that the AI carries allow for a more informed practitioner action and more specific, AI-tailored development of the implementation methodologies.

## Data Availability

The datasets presented in this study can be found in online repositories. The names of the repository/repositories and accession number(s) can be found in the article/Supplementary Material.
