# Supplementary material for: Critical activities for successful implementation and adoption of AI in healthcare: towards a process framework for healthcare organizations
Source: Front Digit Health. 2025 May 16;7:1550459. doi: 10.3389/fdgth.2025.1550459 (PMC12122488; doi:10.3389/fdgth.2025.1550459)
Supplement: Supplementary file 1 [file Datasheet1.pdf]

Supplementary Material S1. Articles' categorization

**AI application**	**Category**	**Article reference**
Digital Pre-Hospital Triage for COVID-19	Diagnosis	Lai et al. (2020) [38]
Sepsis Detection using AI Algorithm	Diagnosis	Gonçalves et al. (2020) [50]
Intracranial Hemorrhage Detection (CT)	Diagnosis	Davis et al. (2020) [47]
NLP for CDS including silent brain infarction and cardiac sarcoidosis	Diagnosis	Wen et al. (2019) [54]
Sepsis Prediction (Hospital and ICU)	Diagnosis	McCoy & Das (2017) [58]
AI DSS for Emergency Care	Diagnosis	Petitgand et al. (2020) [51]
Sepsis Detection with Sepsis Watch	Diagnosis	Sendak et al. (2020) [13]
AI for Radiology Diagnostics	Diagnosis	Strohm et al. (2020) [44]
CDS for Glycemic Control in Diabetes	Diagnosis	Romero-Brufau et al. (2020) [48]
Predictive Analytics for Patient Monitoring	Diagnosis	Moorman (2021) [49]
AI for Language Translation in Radiology	Diagnosis	Chonde et al. (2021) [62]
Voice-based EMR, medical image, chatbot, and diagnostic AI	Diagnosis	Sun et al. (2021) [63]
Automatic Video Auditing (Hand Hygiene)	Prevention	Lacey et al. (2020) [59]
Mission Control for Patient Flow	Prevention	Schlicher et al. (2021) [56]
AI-Enhanced TB Screening	Prevention	Herman et al. (2022) [53]
Emotional Support via AI Chatbot	Treatment	Joerin et al. (2019) [61]
Clinical Pathway Adherence using AI	Treatment	Cruz et al. (2019) [60]
Emergency Department Workflow Optimization	Treatment	Lee et al. (2015) [66]
Unplanned Readmission Prediction	Treatment	Baxter et al. (2020) [45]
Wireless Monitoring System for Vitals	Treatment	Xu et al. (2020) [57]
Clinical Decision Support for Readmission Risk	Treatment	Romero-Brufau et al. (2020) [48]

AI for Sepsis Treatment (Sepsis Watch)	Treatment	Sendak et al. (2020) [41]
Individual-Centric Hospital Discharge	Treatment	Ng & Tan (2021) [46]
Clinical Decision Support for Neonatal Care	Treatment	Wijnhoven (2022) [43]
Predictive Model for Palliative Care	Treatment	Murphree et al. (2021) [55]
Digital Platform for Rehabilitation	Treatment	Saverino et al. (2021) [52]
Reducing nonbillable activities of physicians	Treatment	Reis et al. (2020) [40]
AI for Early Detection and Prevention of Sepsis	Treatment	Sandhu et al. (2020) [42]
Analytical projects at Lucile Packard Hospital	Treatment	Sheinker et al. (2020) [39]
