# Supplementary material for: Critical activities for successful implementation and adoption of AI in healthcare: towards a process framework for healthcare organizations
Source: Front Digit Health. 2025 May 16;7:1550459. doi: 10.3389/fdgth.2025.1550459 (PMC12122488; doi:10.3389/fdgth.2025.1550459)
Supplement: Supplementary file 2 [file Datasheet2.pdf]

Supplementary Material S2. Interview study II guide

Questions to guide the interviews with experts about the Quality Implementation Framework when used in connection with the implementation of Artificial Intelligence.

1. In your opinion, is there a need for any kind of guidance or structural support to facilitate the implementation of different AI applications in healthcare? Would that guidance be different for different kinds of AI or does the need for guidance have a more generic character?
2. Which are the potential benefits of a guide that points out important decisions/actions/activities in connection with initiating and using different AI applications in healthcare? Are there any potential disadvantages, and which would these be?
3. What is your impression of the Quality Implementation Framework in connection with the implementation of AI? Is the guide too generic or too specific? Too general or too detailed? Any other opinions on aspects in the micro-level of the framework?
4. Do you see any important aspects missing in the framework? What are they and why are they needed? Are there any superfluous parts that are not important or relevant in your opinion? Which would these be and why?
