# Supplementary material for: Critical activities for successful implementation and adoption of AI in healthcare: towards a process framework for healthcare organizations
Source: Front Digit Health. 2025 May 16;7:1550459. doi: 10.3389/fdgth.2025.1550459 (PMC12122488; doi:10.3389/fdgth.2025.1550459)
Supplement: Supplementary file 3 [file Datasheet3.pdf]

Supplementary Material S3. Mapping of data against Quality Implementation Framework (QIF)

Literature	Interview study I: Experts on QIF	Interview study II: Cases	Identified extra steps or changes needed	QIF step relevant to implementation of AI?
**PHASE 1. INITIAL CONSIDERATIONS REGARDING THE HOST SETTING**				
**QIF Step 1. Conducting a needs and resource assessment**<br>A. Why are we doing this?<br>B. What problems or conditions will the innovation address (i.e., the need for the innovation)?<br>C. What part(s) of the organization and who in the organization will benefit from improvement effort?				
• Performing analysis by using data to understand the root cause and magnitude of the specific problem and where it occurs in the specific hospital (13, 38-42)	• Finding the right data for the problem (Expert 1)<br>• Identifying the patient perspective corresponding to the needs (Expert 2)	• Formulating a problem (Case 1, Case 2, Case 3, Case 4)<br>• Creating a case demonstrating a clear need: what should be solved and why (Case 2)<br>• Performing internal studies showing the problem to motivate the need, and external studies to explain that it is possible to solve the need by using AI software (Case 2)	• Guiding how to perform the needs analysis (Expert 4)	Yes
**QIF Step 2. Conducting a fit assessment**<br>A. Does the innovation fit the setting?<br>B. How well does the innovation match the: B1. Identified needs of the organization/community? B2. Organization's mission, priorities, values, and strategy for growth? B3. Cultural preferences of group/consumers who participate in activities/services provided by the organization/community?				
• Checking alignment of AI implementation with the innovation strategy and institutional priorities,	• Understanding whether the intended application suits and solves the needs (Expert 1, Expert 3)	• Exploring of a fitting commercial solution on the market (Case 1, Case 2, Case 3)	• Assessing compatibility of old processes with new tools (46)	Yes

preferences and needs of clinicians and other stakeholders as well as expectations for evidence<br>• Identifying potential added value whether AI would improve clinical practice or achieve operational effectiveness<br>(39-40, 43-45)	• Understanding whether the use of AI can/will lead to the ability for the organization to deliver care/benefit, which cannot be provided today (Expert 2)		• Investigating if the product has the right certification (Expert 3, Case 3)<br>• Investigating relevance of the data that the model was built on (Expert 3, Expert 6)<br>• Investigating what are the conditions for retraining the model and for monitoring performance (Expert 6)<br>• Performing a Data Protection Impact Assessment (Case 3)<br>• Investigating ethical aspects of the solution: bias, participation, integrity, demographics, etc. (Expert 3)<br>• User needs guidance on what legal aspects of the AI solution and of collaboration with vendors need to be assessed (Expert 4, Case 3)<br>• Performing a risk-consequence analysis identifying benefits and risks to patients (Expert 2, Case 4)	
**QIF Step 3. Conducting a capacity/readiness assessment**<br>A. Are we ready for this?<br>B. To what degree does the organization/community have the will and means (i.e., adequate resources, skills, and motivation) to implement the innovation?<br>C. Is the organization/community ready for change?				

• Identifying resources needed (13, 39, 43, 45)		• Analysing whether other jobs could be offered to the staff that would lose the job due to AI (Case 4)	• Performing a cost-benefit analysis (43)<br>• Considering implementation cost (Case 2)<br>• Investigating the opportunity cost: what might be lost due to the introduction of AI, e.g. deskilling staff (Expert 3)<br>• Investigating sufficiency of the technical environment in an organization (e.g. computing power) (Expert 6)	Yes
**QIF Step 4. Possibility for adaptation**<br>A. Should the planned innovation be modified in any way to fit the host setting and target group?<br>B. What feedback can the host staff offer regarding how the proposed innovation needs to be changed to make it successful in a new setting and for its intended audience?<br>C. How will changes to the innovation be documented and monitored during implementation?<br>Capacity Building Strategies (may be optional depending on the results of previous elements)				
• Identifying constraints<br>• Viewing AI system in the context of other data sources and systems at the organization<br>• Deciding if any additional developments in IT, infrastructure, workflows might be needed<br>(13, 39, 43-49)		• Investigating AI system's compatibility with existing supported systems and hardware units (Case 4)<br>• Setting up a local pilot to identify local relevance and required adaptations (Case 1, Case 3, Case 4)		Yes
**QIF Step 5. Obtaining explicit and implicit buy-in and approvals/permissions**				

5A. Do we have genuine and explicit buy-in for this innovation from: * Leadership with decision-making power in the organization/community? * Front-line staff who will deliver the innovation? * The local community (if applicable)?<br><br>5B. Have we effectively dealt with important concerns, questions, or resistance to this innovation? What possible barriers to implementation need to be lessened or removed?<br><br>5C. Can we identify and recruit an innovation champion(s)? * Are there one or more individuals who can inspire and lead others to implement the innovation and its associated practices? * How can the organization/community assist the champion in the effort to foster and maintain buy-in for change?<br><br>Note: Fostering a supportive climate is also important after implementation begins and can be maintained or enhanced through such strategies as organizational policies favoring the innovation and providing incentives for use and disincentives for non-use of the innovation				
• Organizing for support from leadership, professional organizations, and analytics-minded clinicians<br>• Showing the added value by the AI system and what value by physicians remains<br>• Appointing local champions to overcome resistance by colleagues<br>(13, 39-41, 44-45, 48-55)	• Evaluating whether the use of AI could enable the delivery of care that is currently unattainable (Expert 3)	• Having a determined specialist – a clinician communicating a need for a solution forward (Case 1, Case 2)<br>• Presenting potential benefits of AI system to colleagues to gain their interest (Case 1, Case 2, Case 4)<br>• Receiving support from leadership (Case 1, Case 2, Case 4)<br>• Appointing local champions to transfer practice to colleagues (Case 3, Case 4)	• Addressing algorithm's explainability, availability, quality and safety (45, 48)<br>• Addressing clinicians' legal liability questions (40, 44), (Case 1, Case 2, Expert 3)<br>• Presenting a thoughtful framing of AI in communication (13)<br>• Communicating trustworthiness, utility of the AI system, and a possibility of turning off the system if improvements are not reached (41)<br>• Involving clinicians in designing a user-friendly AI system and user interface, in system's training and in contextualizing data representation (41)	Yes
**QIF Step 6. Building organizational capacity**<br><br>6A. What infrastructure, skills, and motivation of the organization/community need enhancement in order to ensure the innovation will be implemented with quality?				

Note: This type of capacity does not directly assist with the implementation of the innovation, but instead enables the organization to function better in a number of its activities (e.g., improved communication within the organization and/or with other agencies; enhanced partnerships and linkages with other agencies and/or community stakeholders).				
• Building relationships that would enable the workflows related to AI system (42)				Yes
**QIF Step 7. Staff recruitment / maintenance**<br>7A. Who will implement the innovation? Initially, those recruited do not necessarily need to have knowledge or expertise related to use of the innovation; however, they will ultimately need to build their capacity to use the innovation through training and on-going support<br>7B. Who will support the practitioners who implement the innovation? These individuals need expertise related to (a) the innovation, (b) its user, (c) implementation science, and (d) process evaluation so they can support the implementation effort effectively<br>7C. Might roles of some existing staff need realignment to ensure that adequate person-power is put towards implementation?				
• Recruiting project leaders and teams<br>• Re-aligning roles of existing staff<br>(13, 40, 42, 56)	• Investigating potential changes to roles and work activities (Expert 3)		• Recruiting staff for managing routine tasks involving AI system (40, 42)	Yes
**QIF Step 8. Effective pre-innovation staff training**<br>8A. Can we provide sufficient training to teach the why, what, when, where, and how regarding the intended innovation?<br>8B. How can we ensure that the training covers the theory, philosophy, values of the innovation, and the skill-based competencies needed for practitioners to achieve self-efficacy, proficiency, and correct application of the innovation?				
• Performing pre- innovation training, as well as building an understanding of how to assess the validity of AI output<br>• Building skills in communication, empathy, attentive listening, and		• Performing training (Case 1, Case 2, Case 4)<br>• Preparing the training materials and an information package about the AI system (Case 1, Case 2, Case 4)	• Performing training during the implementation, focusing on the vision for change and skills of using the AI solution (13, 42, 48, 49)	Yes

technical knowledge about the AI system<br>• Providing lectures, demonstrations, online training<br>(13, 39, 42, 48-50, 56-58)			• Performing post-implementation training focusing on analysis of concrete cases (39, 49)<br>• Preparing formalized process flowcharts for the users to follow (56).<br>• Costs of preparing and communicating training material should be considered (13)	
**PHASE 2: CREATING A STRUCTURE FOR IMPLEMENTATION**				
**QIF Step 9. Creating implementation teams**<br>A. Who will have organizational responsibility for implementation?<br>B. Can we develop a support team of qualified staff to work with front-line workers who are delivering the innovation?<br>C. Can we specify the roles, processes, and responsibilities of these team members?				
• Recruiting staff to the AI implementation project (13, 40, 56)	• Developing the necessary competencies for AI implementation, including medical expertise, IT and technical proficiency, business-oriented skills, change management capabilities, and the redefinition of staff roles. (Expert 7)	• Appointing or recruiting staff to the AI implementation project (central project management and regional) (Case 1, Case 2, Case 4)		Yes
**QIF Step 10. Developing an implementation plan**<br>A. Can we create a clear plan that includes specific tasks and timelines to enhance accountability during implementation?<br>B. What challenges to effective implementation can we foresee that we can address proactively?				
• Setting goals for the ex-ante and ex-post implementation.			• Developing a communication plan (Case 4)	Yes

<table>
<tr><td>• Planning a 3-months silent period before the launch during which AI model can interact with real-time clinical data.
• Developing meeting plan prior to launch to cultivate relationships and communication channels between the roles
(13, 44)</td><td></td><td></td><td>• Deciding on the organizational owner of the AI system (Case 2)</td><td></td></tr>
<tr><td colspan="5">PHASE 3: ONGOING STRUCTURE ONCE IMPLEMENTATION BEGINS</td></tr>
<tr><td colspan="5">QIF Step 11. Technical assistance/coaching/supervision governance
A. Can we provide the necessary technical assistance to help the organization/community and practitioners deal with the inevitable practical problems that will develop once the innovation begins?
Note: These problems might involve a need for further training and practice in administering more challenging parts of the innovation, resolving administrative or scheduling conflicts that arise, acquiring more support or resources, or making some required changes in the application of the innovation.</td></tr>
<tr><td>• Providing engineer support and peer support to the team throughout the implementation
• Equipping staff with hardware, software, training materials, contact information
(13, 52)</td><td></td><td>• Providing team chat, IT support and FAQ on Intranet (Case 4)
• Providing technical and clinical support by project leaders (Case 1)</td><td></td><td>Yes</td></tr>
<tr><td colspan="5">QIF Step 12. Process evaluation
A. Do we have a plan to evaluate the relative strengths and limitations in the innovation’s implementation as it unfolds over time?
Note: Data are needed on how well different aspects of the innovation are being conducted as well as the performance of different individuals implementing the innovation.</td></tr>
</table>

• Collecting users' quantitative and qualitative feedback to trigger improvements in product, workflow, user interface (13, 61)<br>• Monitoring users' engagement and usage (59)<br>• Evaluating clinical and operational, and process impact to demonstrate added value, safety and efficacy (13, 39, 44, 48, 60)Forming partnerships with research institutions for evaluating the project from various angles, e.g. organizational change in the context of AI, adoption barriers and facilitators (13)<br>(13, 39, 44, 48, 59-61)	• Evaluation and its methods should be considered right at the beginning when looking at the data and defining what problem will be solved (Expert 1)	• Setting KPIs for users' engagement and usage (Case 4)<br>• Monitoring different measures of the system's performance (Case 1, Expert 6)<br>• Performing retrospective studies on effects of AI and impact on patient management (Case 1)		Yes
**QIF Step 13. Supportive feedback mechanism**<br>13A. Is there an effective process through which key findings from process data related to implementation are communicated, discussed, and acted upon?<br>13B. How will process data on implementation be shared with all those involved in the innovation (e.g., stakeholders, administrators, implementation support staff, and front-line practitioners)?<br>Note: This feedback should be offered in the spirit of providing opportunities for further personal learning and skill development and organizational growth that leads to quality improvement in implementation				
• Collecting clinical feedback after deployment<br>• Providing web-based survey or e-mail for continuous feedback from frontline staff including	• Providing easy ways for staff and patients to point out things that do not work and risks (Expert 3)	• Providing regular check-ins with key staff (Case 1)	• Forming an AI governance committee to continuously monitor model's effectiveness, promote usage, provide training, develop reporting, plan for	Yes

doubts, training requests, suggestions for improvement<br>• Performing regularly scheduled feedback meetings post-implementation<br>• Setting prizes to the users who can contribute to the AI system's usage or improvement<br>(13, 41, 45, 50, 57, 58, 62, 63)			sustainability of the system (13, 41, 45), (Expert 6)<br>• Monitoring for a proper insertion of records from the patients, and eventual difficulties in the use of the tool by the end-users (50)	
**PHASE 4: IMPROVING FUTURE APPLICATIONS**				
**QIF Step 14. Learning from experience**<br>14A. What lessons have been learned about implementing this innovation that we can share with others who have an interest in its use?				
	• Providing continuous maintenance and development of the AI system to fit the business<br>• Transferring knowledge about how to continuously improve: especially important if external consultants have been used to assist in the implementation (Expert 1)			Yes
